# Antioxidants Reduce Muscular Dystrophy in the *dy^2J^/dy^2J^* Mouse Model of Laminin α2 Chain-Deficient Muscular Dystrophy

**DOI:** 10.3390/antiox9030244

**Published:** 2020-03-18

**Authors:** Vahid M. Harandi, Bernardo Moreira Soares Oliveira, Valérie Allamand, Ariana Friberg, Cibely C. Fontes-Oliveira, Madeleine Durbeej

**Affiliations:** 1 Unit of Muscle Biology, Department of Experimental Medical Science, Lund University, 221 84 Lund, Sweden; bernardomso@bmb.sdu.dk (B.M.S.O.); valerie.allamand@inserm.fr (V.A.); ariana.friberg.302@student.lu.se (A.F.); ccristine@gmail.com (C.C.F.-O.); madeleine.durbeej-hjalt@med.lu.se (M.D.); 2 Functional Genomics & Metabolism Unit, Department of Biochemistry & Molecular Biology, University of Southern Denmark, 5230 Odense, Denmark; 3 Centre de Recherche en Myologie, Sorbonne Université, Inserm, UMRS974, 75013 Paris, France

**Keywords:** laminin, reactive oxygen species, congenital muscular dystrophy, therapy

## Abstract

Congenital muscular dystrophy with laminin α2 chain-deficiency (LAMA2-CMD) is a severe neuromuscular disorder without a cure. Using transcriptome and proteome profiling as well as functional assays, we previously demonstrated significant metabolic impairment in skeletal muscle from LAMA2-CMD patients and mouse models. Reactive oxygen species (ROS) increase when oxygen homeostasis is not maintained and, here, we investigate whether oxidative stress indeed is involved in the pathogenesis of LAMA2-CMD. We also analyze the effects of two antioxidant molecules, N-acetyl-L-cysteine (NAC) and vitamin E, on disease progression in the *dy^2J^/dy^2J^* mouse model of LAMA2-CMD. We demonstrate increased ROS levels in LAMA2-CMD mouse and patient skeletal muscle. Furthermore, NAC treatment (150 mg/kg IP for 6 days/week for 3 weeks) led to muscle force loss prevention, reduced central nucleation and decreased the occurrence of apoptosis, inflammation, fibrosis and oxidative stress in LAMA2-CMD muscle. In addition, vitamin E (40 mg/kg oral gavage for 6 days/week for 2 weeks) improved morphological features and reduced inflammation and ROS levels in *dy^2J^/dy^2J^* skeletal muscle. We suggest that NAC and to some extent vitamin E might be potential future supportive treatments for LAMA2-CMD as they improve numerous pathological hallmarks of LAMA2-CMD.

## 1. Introduction

Congenital muscular dystrophy type 1A (LAMA2-CMD) is a severe, recessive autosomal form of muscular dystrophy. The disease is characterized by muscle hypotonia, progressive muscle degeneration and muscle weakness. Other clinical hallmarks of LAMA2-CMD include proximal joint contractures, scoliosis and respiratory insufficiency. As a result, patients experience a decreased quality of life and most often the disease also leads to premature death. LAMA2-CMD is caused by mutations in the *LAMA2* gene, encoding the laminin α2 chain of the protein laminin-211 [1]. Laminin-211 is one of the major components expressed in the skeletal muscle basement membrane [2] and the interaction between laminin-211 and integrin and dystroglycan receptors provides a linkage between the basement membrane and the actin cytoskeleton. This linkage is of high importance for normal skeletal muscle function as it stabilizes the sarcolemma and protects the muscle fiber from contraction-induced damage [3]. As a consequence, when this important linkage is broken, a typical dystrophic pattern becomes evident. Fiber size variation, centrally located nuclei, inflammation and fibrotic lesions are common features that characterize LAMA2-CMD muscles [1].

There are several mouse models that adequately recapitulate LAMA2-CMD [4,5,6,7]. The *dy^2J^/dy^2J^* mouse carries a mutation in the N-terminal domain of laminin α2 chain causing a laminin polymerization defect and slightly reduced expression of the laminin α2 chain lacking this domain [4]. Accordingly, *dy^2J^/dy^2J^* mice develop a milder form of muscular dystrophy with the first symptoms appearing at around 3–4 weeks and a longer life span compared to other mouse models. In addition, a severe peripheral neuropathy manifests in *dy^2J^/dy^2J^* mice [1,4,5,6,7,8].

We have previously performed transcriptional and proteomic profiling of LAMA2-CMD mouse muscles and found that a majority of the dysregulated genes and proteins are involved in various metabolic processes, indicating a metabolic crisis in LAMA2-CMD muscles [9,10]. More recently, a metabolic impairment, with reduced mitochondrial respiration and enhanced glycolysis was observed in human laminin α2-chain deficient muscle cells [11]. Insufficient mitochondrial respiration in turn, enhances the formation of reactive oxygen species (ROS), which long have been suggested to be major contributors to muscle damage in dystrophic muscles [12]. However, whether ROS levels are augmented in LAMA2-CMD remains to be determined.

Given the potentially important effect of ROS on muscle damage in dystrophic muscles, antioxidant treatment to reduce the oxidative stress has been postulated as a promising approach to improve muscle health [13,14]. N-acetylcysteine (NAC) is a compound with strong antioxidant properties. In addition to directly functioning as a scavenger of ROS, NAC also acts indirectly as an antioxidant (as a precursor to the amino acid cysteine that is required for the biosynthesis of the cellular antioxidant glutathione) [15]. Importantly, NAC is considered a safe drug and has long been used to treat acetaminophen overdose and to thin out mucus in individuals with cystic fibrosis [16,17]. NAC is also emerging as a treatment for a wide range of medical conditions including psychiatric and neurological disorders [18]. Vitamin E is another compound with antioxidant activity. It has been demonstrated to promote plasma membrane repair acting as a membrane-based antioxidant [19]. Furthermore, it has been shown to have a close relationship with muscle health as vitamin E-deficiency is associated with muscle weakness, loss of muscle strength and myopathy [20,21].

The aim of the present study was to determine whether ROS levels are increased in LAMA2-CMD muscle and to evaluate the possible protective roles of NAC and vitamin E, respectively, against ROS-induced muscle damage in *dy^2J^/dy^2J^* mice. More specifically, we analyzed the effects of NAC and vitamin E on muscle strength, muscle morphology, apoptosis, inflammation, fibrosis and ROS levels.

## 2. Materials and Methods 

### 2.1. Animals

Heterozygous *dy^2J^/dy^2J^* (B6.WK-*Lama2*dy-2J/J) mice were obtained from Jackson Laboratory and bred and maintained in the Biomedical Centre, Lund University animal facility according to institutional animal care guidelines. All experimental procedures involving animals were approved by the Malmö/Lund (Sweden) ethical committee for animal research (ethical permit number 5.8.18-02255/2017 and 5.8.18-05195/2018) in accordance with guidelines issued by the Swedish Board of Agriculture. The animals were maintained at 22 ± 2 °C with a regular light-dark cycle (light on from 6:00 am to 6:00 pm) and had free access to food and water. The diet consisted of 51.2% carbohydrate, 22% protein and 4.25% fat (Special Diet Services). Three-week-old mice were subdivided into wild-type (WT) control, WT NAC-treated, *dy^2J^/dy^2J^* control and *dy^2J^/dy^2J^* NAC-treated. Similarly, 3-week-old mice were subdivided into WT control, WT vitamin E-treated, *dy^2J^/dy^2J^* control and *dy^2J^/dy^2J^* vitamin E-treated.

### 2.2. Human Tissues

Different muscles from both control individuals (age ranged between 2.5 months to 33 years) and patients (age ranged between 22 days to 29 years) were biopsied (see Table 1). Cryo-sections were kindly provided by Maud Beuvin and Dr Norma B Romero from the Neuromuscular Morphological Unit (Myology Institute, Paris, France).

### 2.3. Treatments

NAC (A9165 Sigma-Aldrich, Saint Louis, MO, USA) was dissolved in saline (0.9%) and administrated by intraperitoneal injections six times a week at 150 mg/kg body weight for 22 days. In addition, control animals received standard control solution (0.9% saline) for the same duration of time. Vitamin E 100 mg dl-α-tocopherol acetate (Meda AB, Solna) was administrated by oral gavage six times a week at 40 mg/kg body weight for 14 days. Control animals received standard control solution (0.9% saline) by oral gavage for 14 days. Initial body weights were recorded at the beginning of the treatment and final body weights were recorded before the animals were sacrificed. 

### 2.4. Stand Ups and Grip Strength

After the last day of treatment, stand-ups and grip strength analyses were performed. Each mouse was placed into a new cage and allowed to explore the cage for five minutes and the number of full stand-ups on hindlimbs were counted for each individual animal.

Forelimb grip strength was measured on a grip-strength meter (Columbus Instruments, Columbus, OH, USA) as previously described [22]. In short, each mouse was held by the base of the tail and allowed to grasp the horizontal pull bar with its forepaws and then each mouse was gently pulled away by its tail until it released the pull bar. The test for each animal was repeated 5 times with 30 s rest in between each measurement. The two lowest values were rejected and the mean of the three highest values was counted. Animals were not subjected to any training prior to the experiment. Normalized grip strength was calculated as force divided by final body weight [23].

### 2.5. Tissue Collection

Mice were sacrificed by cervical dislocation. Thereafter, tissues (quadriceps, triceps and diaphragm muscles and liver) were carefully dissected and weighed.

### 2.6. Histology and Immunohistochemistry

For morphometric analyses, quadriceps and triceps muscles were either embedded in OCT compound (Tissue-Tek, Torrance, CA, USA) and frozen in liquid nitrogen or embedded in paraffin. Paraffin-embedded specimens were sectioned using a microtome (5 μm) (Microm H355) and OCT embedded sections were sectioned using a cryostat (7 μm) (Microm HM 560). Paraffin sections were stained with hematoxylin and eosin (H&E) and cryo-sections were subjected to immunostaining. Thereafter, H&E-stained cryo-sections were scanned using an Aperio ScanScope CS2 scanner with ScanScope console version 8.2.0.1263. Central nucleation was quantified using ImageJ software version 1.43u, Cell Counter plug-in (NIH). The percentage of central nuclei was calculated by counting all the fibers in quadriceps and triceps cross-sections.

In addition, the cross-sectional area of WGA-stained muscle fibers was measured using Image J on cryo-sections of stained muscle fibers. At least 1000 fibers per quadriceps and triceps cross-sections were analyzed.

Immunohistochemistry was performed as previously described [24] using a monoclonal antibody against CD11b (rat monoclonal M1/70,1:250, BD Pharmingen, San Jose, CA, USA), anti-4 hydroxynonenal (4HNE) antibody (rabbit polyclonal ab46545, 1:200, Abcam, Cambridge, United Kingdom) and caspase-3 antibody (mouse monoclonal, 1/100, CPP32, BD Transduction Laboratory) on cryo-sections. To visualize the cell membrane, biotinylated WGA or laminin γ1 chain antibody (rat monoclonal 1/200, MAB 1914, Chemicon, Darmstadt, Germany) were used. The secondary antibody was goat anti-mouse IgG 546 (Thermo Fisher Scientific) for CD11b and donkey anti-rabbit IgG 488 (Thermo Fischer Scientific, San Diego, CA, USA) for 4HNE. The slides were analyzed by Zeiss Axioplan fluorescence microscope (Zeiss, Oberkochen, Germany) and images were captured using an ORCA 1394 ER digital camera (Hamamatsu Photonics, Hertfordshire, United Kingdom) and Openlab software version 4 (Improvision, Coventry, United Kingdom). For quantification of CD11b and 4HNHE immunostaining, ImageJ software (1, 52q, National Institute of Health, USA) was used. The area corresponding to CD11b or 4HNE labelling was quantified relative to the entire area of the cross-section.

### 2.7. Morphometric Evaluation of Fibrosis by Fast Green and Sirius Red Staining

For collagen quantification, quadriceps and triceps cryo-sections (8 µm) were stained with Fast Green and Sirius Red dye combination. Sirius Red binds selectively to fibrillar collagens, whereas Fast Green binds to non-collagenous proteins, making it easy to distinguish collagen fibers from other non-collagenous proteins. Firstly, slides were fixed for 1 h in Bouins Solution at 55 °C, stained with Fast Green (10 min at room temperature), dipped 10 times in distilled water and then incubated in 0.1% Picro Sirius Red for 30 min (Sigma-directed red 80 in saturated aqueous picric acid, Saint Louis, MO, USA). Slides were then dehydrated in three changes of increasing ethanol concentrations (70, 95 and 100%) for 2 min per change. Lastly, slides were cleared in xylene (2 min) and coverslips were mounted onto the slides using Pertex mounting medium (Histolab, 00840, Gothenburg, Sweden). Sirius Red positive areas were quantified using ImageJ and presented as percentage of total section area.

### 2.8. Colorimetric Evaluation of Fibrosis by Fast Green/Sirius Red Staining

As Fast Green and Sirius Red, respectively, absorb light at different wavelengths, the optical density (OD) of the extracted dyes can be used to calculate the collagen/protein ratio of the sample. The quantification procedure was performed according to the manufacturer’s protocol. In summary, paraffin-embedded quadriceps and triceps muscles were cut into sections (15 µm) and were then transferred to a 5 mL tube. Sections were deparaffinized after being incubated in xylene (5 min), xylene: ethanol (1:1) (5 min), 100% ethanol (5 min), water: ethanol (1:1) (5 min) and water (5 min). The water was removed and sections were incubated in 0.1% Fast Green and 0.1% Sirius Red for 30 min under rotation at room temperature. The fluid was then removed and the sections were rinsed several times until the rinsing fluid became colorless. The stains were then eluted by adding a volume of 1:1 mixture of NaOH (0.1 N) and 100% methanol for 2 min. The eluted dyes were transferred to a 96-wellplate and the absorbance was read at 540 nm (red) and 605 nm (green) by a spectrophotometer. The amount of collagen and non-collagenous proteins was then calculated. The corrected absorbance was calculated by subtracting the value corresponding to 29.1% of the OD at 605 nm from the absorbance 540 nm. In order to measure the collagen and non-collagenous protein, the absorbance at 605 nm and the corrected absorbance at 540 nm were divided by their respective color equivalence (2.08 and 38.4).

### 2.9. Dihydroethidium Staining for ROS Detection

Dihydroethidium (DHE), a commonly used indicator of ROS production is oxidized by ROS, forming ethidium that fluoresces red when intercalated with DNA. To determine the levels of ROS, muscle sections were incubated with DHE (D7008, Sigma-Aldrich, Saint Louis, MO, USA) for 30 min at 37 °C. In brief, 5 mM DHE was applied to the muscle sections and thereafter *in situ* fluorescence was assessed using fluorescence microscopy. DHE staining was quantified by measuring pixels exceeding a specified threshold, which was set in order to eliminate interference from any background fluorescence. The whole cross-section area was used for quantification and the percentage of area with positive staining was calculated. 

### 2.10. Real Time PCR Analysis

RNA isolation was performed by using RNeasy Fibrous Tissue Kit QIAGEN according to manufacturer’s recommendations. First-strand cDNA was synthesized from total RNA (0.8 μg) with oligonucleotide (dT)15 primers and random primers p(dN)6 by use of First Strand cDNA synthesis kit (Roche, Mannheim, Germany). Real time-PCRs were performed using Light Cycler 480 SYBR Green Master I (Roche, Mannheim, Germany) and were analyzed by Light Cycler 480 SW 1.5 software (Roche, Mannheim, Germany). Primers used in our experiments were from Sigma (KiCqStart SYBR Green Primers, Saint Louis, MO, USA). Amplification conditions consisted of 5 s of denaturation at 94 °C, 9 s of annealing at 55–60 °C and 9 s of extension at 72 °C for each step for 45 cycles. The relative amount of all mRNAs was calculated using the comparative CT method (ΔΔCt). *Rplp0* was used as the invariant control [25].

### 2.11. Statistical Analysis and Data Availability

All statistical analyses were performed with GraphPad Prism software version 8. All experimental data are presented as means ± S.E.M. Statistical analysis of the data were performed by mean of one-way analysis of variance (ANOVA) using non-parametric test with Dunn’s post hoc test for comparison of treatment effects when comparing differences between more than two groups or by Mann–Whitney unpaired t-test when only two groups were used for an experiment. Statistical significance was considered for p values lower than 0.05. The datasets generated during and/or analyzed during the current study are available from the corresponding author on reasonable request.

## 3. Results

### 3.1. Significantly Increased ROS Levels in Skeletal Muscles of dy^2J^/dy^2J^ Mice and LAMA2-CMD Patients

**Figure 1:**
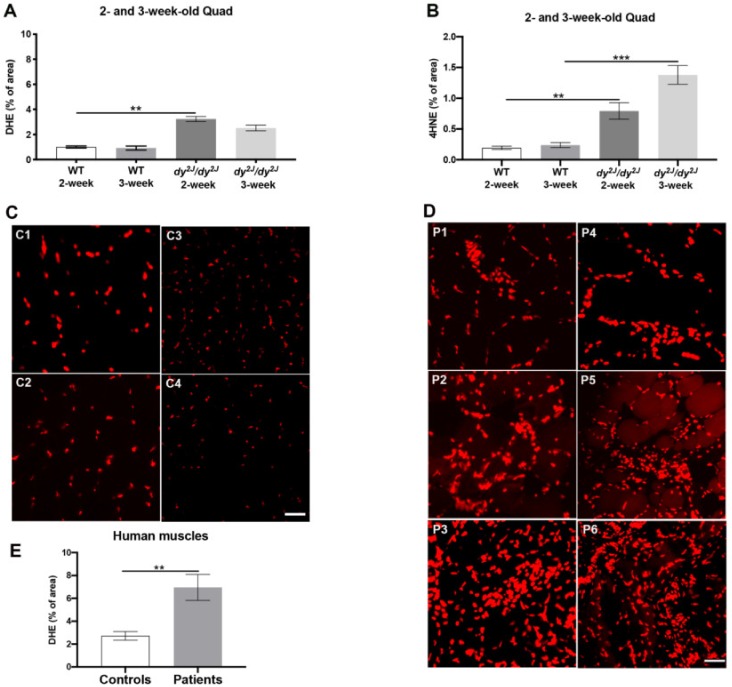
(**A**,**B**): Dihydroethidium (DHE)- and 4-hydroxynonenal (4HNE)-positive areas in quadriceps muscles of 2- and 3-week-old animals; (**C**) Representative DHE staining of four control individuals (C_1_–C_4_); (**D**) Representative DHE staining of the six patients (P_1_–P_6_); (**E**) DHE-positive areas in four control individuals and six patients. Results are expressed as mean ± SEM in 5 WT and 6 *dy^2J^/dy^2J^* (DHE staining, 2-week-old mice), 4 WT and 6 *dy^2J^/dy^2J^* (DHE staining, 3-week-old mice), 4 WT and 4 *dy^2J^/dy^2J^* (4HNE staining, 2-week-old animals), 5 WT and 5 *dy^2J^/dy^2J^* (4HNE staining 3-week-old animals). ** *p* < 0.01, *** *p* < 0.001. Bar: 50 μm.

To analyze ROS levels in *dy^2J^/dy^2J^* muscle at early stages of disease, we used dihydroethidium (DHE) and an antibody against 4-hydroxynonenal (4HNE). DHE is oxidized by ROS, forming ethidium that fluoresces when intercalated with DNA while 4HNE is a marker for lipid peroxidation and thus detects ROS-caused alteration of macromolecules. We found significantly enhanced ROS levels in 2- and 3-week-old *dy^2J^/dy^2J^* quadriceps muscle (Figure 1A,B, Supplementary Material Figure S1). We observed that the brightest positive areas in *dy^2J^/dy^2J^* quadriceps and triceps muscles occurred in densely packed areas, most likely corresponding to infiltrating inflammatory cells as these cells also produce significant amounts of ROS [26].

ROS levels were also higher in LAMA2-CMD patient muscle biopsies compared with control individuals’ muscle where the percentage of DHE-positive areas was lower (Table 1, Figure 1C–E). In biopsies from patients’ number 2, 3, 5 and 6, all with complete laminin α2-deficiency, we observed a larger DHE-positive area as opposed to those from patients’ number 1 and 4 with partial laminin α2-deficiency (Table 1, Figure 1D).

To summarize, we demonstrate that ROS levels are significantly augmented in LAMA2-CMD skeletal muscles.

### 3.2. Muscle Force Loss is Prevented by NAC Treatment

Since the production of ROS was increased in mouse and human LAMA2-CMD muscles we envisaged that antioxidant treatment could improve muscle function and morphology. Due to the fact that the first symptoms of *dy^2J^/dy^2J^* mice appear at around three weeks of age [8,27], we decided to treat the animals as early as three weeks to prevent the progression of the disease. Furthermore, Pasteuning-Vuhman *et al.*, recommend starting treatment as soon as possible in the *dy^2J^/dy^2J^* mouse model [8]. Thus, we administered NAC systemically and vitamin E by oral gavage, to 3-week-old *dy^2J^/dy^2J^* animals. NAC treatment continued for 22 days and vitamin E treatment for 14 days. The administrated dose and treatment duration for NAC was based on a number of studies where 150 mg/kg NAC has been used [28,29,30]. For vitamin E administration, we adapted the protocol of Mancio *et al.*, demonstrating that 40 mg vitamin E/kg daily oral gavage for two weeks reduced muscular dystrophy in *mdx* mice [31].

NAC-treated animals were thus 6-week-old at time of sacrifice, while vitamin E-treated animals were 5-week-old. The initial body weights of the different groups (WT, WT NAC, *dy^2J^/dy^2J^*, *dy^2J^/dy^2J^* NAC and WT, WT vitamin E, *dy^2J^/dy^2J^*, *dy^2J^/dy^2J^* vitamin E) were very similar and we did not notice any significant difference in the final body weight between the different groups of mice (Supplementary Material Figure S2).

Nevertheless, we separately analyzed quadriceps, triceps, diaphragm and liver weights after treatment. There was a significant decline in quadriceps and triceps (but not diaphragm) muscle mass in 6-week-old *dy^2J^/dy^2J^* mice and NAC treatment slightly increased the weight of quadriceps muscles (but statistically non-significant). We noticed a significant reduction in liver weight in NAC-treated WT animals but not in NAC-treated *dy^2J^/dy^2J^* mice (Supplementary Material Figure S3A). No significant differences in tissue weights were observed in 5-week-old *dy^2J^/dy^2J^* mice and vitamin E had no further impact on final tissue weight (Supplementary Material Figure S3B).

**Figure 2:**
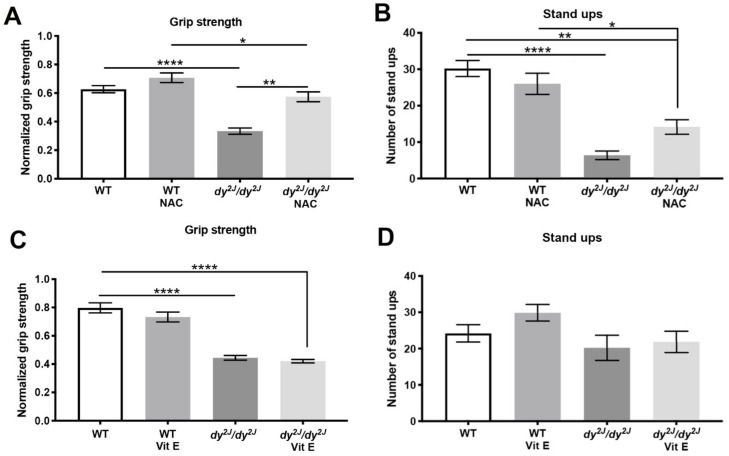
(**A,C**): Normalized forelimb grip strength. Calculations were done by dividing force (KgF) by final body weight in grams. Results are expressed as normalized grip strength in 8 WT, 10 WT N-acetyl-L-cysteine (NAC), 5 *dy^2J^/dy^2J^*, 6 *dy^2J^/dy^2J^* NAC and 11 WT, 8 WT vit E, 5 *dy^2J^/dy^2J^* and 6 *dy^2J^/dy^2J^* vit E; (**B**) Number of stand ups in 10 WT, 11 WT NAC, 5 *dy^2J^/dy^2J^*, 6 *dy^2J^/dy^2J^* NAC and (**D**) 11 WT, 7 WT vit E, 5 *dy^2J^/dy^2J^*, 6 *dy^2J^/dy^2J^* vit E. * *p* < 0.05, ** *p* < 0.01, *** *p* < 0.001 and **** *p* < 0.0001.

As previously described, we observed a decrease in *dy^2J^/dy^2J^* forelimb grip strength at six weeks of age [25,32] (Figure 2A,C). Notably, NAC treatment significantly prevented grip strength reduction in *dy^2J^/dy^2J^* mice (Figure 2A). In contrast, vitamin E did not prevent forelimb grip strength decline compared to untreated *dy^2J^/dy^2J^* mice (Figure 2C). The number of stand-ups was significantly decreased in 6-week-old *dy^2J^/dy^2J^* mice (Figure 2B) but not in 5-week-old *dy^2J^/dy^2J^* mice (Figure 2D). Neither NAC nor vitamin E prevented muscle strength decline that is reflected by the number of stand-ups (Figure 2B,D).

All in all, these data suggest that NAC treatment prevents grip strength decline in *dy^2J^/dy^2J^* muscle. Importantly, neither IP injections of NAC nor oral gavage vitamin E administration affected body weights of animals.

### 3.3. Skeletal Muscle Histology is Preserved by Both NAC and Vitamin E Treatment

**Figure 3:**
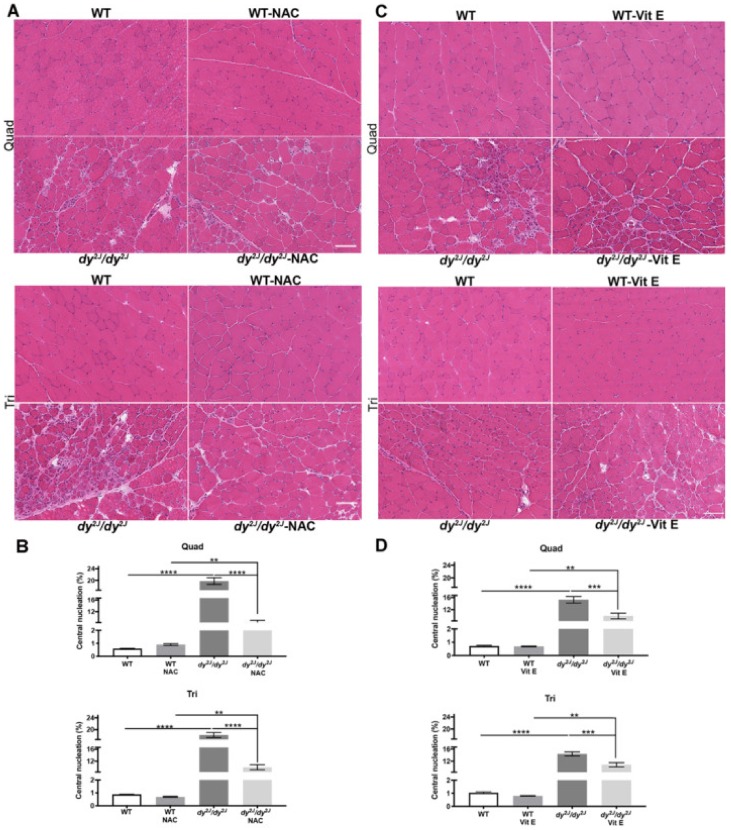
(**A**) Representative hematoxylin and eosin-stained quadriceps and triceps muscle sections (NAC treatment); (**B**) Number of centrally nucleated myofibers in 8 WT, 10 WT NAC, 5 *dy^2J^/dy^2J^*, 6 *dy^2J^/dy^2J^* NAC quadriceps and triceps muscles. Results are expressed as mean ± SEM. (**C**) Representative hematoxylin and eosin-stained quadriceps and triceps muscle sections (vitamin E treatment); (**D**) Number of centrally nucleated fibers in 6 WT, 5 WT vit E, 5 *dy^2J^/dy^2J^*, 5 *dy^2J^/dy^2J^* vit E in quadriceps and triceps muscles. Results are expressed as mean ± SEM. ** *p* < 0.01, *** *p* < 0.001 and **** *p* < 0.0001. Bars: 100 μm.

A specific pattern of muscle involvement has been demonstrated in *dy^2J^/dy^2J^* mice and it has been shown that triceps is less affected compared to hindlimb muscles [8,33]. Therefore, we followed the recommendations of Pasteuning-Vuhman *et al.*, to assess several muscles [8]. The evaluation of H&E-stained *dy^2J^/dy^2J^* quadriceps and triceps sections revealed typical muscular dystrophy characteristics with fiber degeneration/regeneration (evidenced by central nucleation), fiber size variability and mononuclear cell infiltration (Figure 3A,C). The proportion of centrally nucleated myofibers was significantly amplified in 5- and 6-week-old *dy^2J^/dy^2J^* quadriceps and triceps muscles. Remarkably, NAC-treatment as well as vitamin E-treatment significantly reduced the number of fibers with centrally located nuclei (Figure 3A–D).

In 6-week-old *dy^2J^/dy^2J^* quadriceps muscle, the percentage of small fibers (cross sectional areas in the range of 0 to 500 μm^2^) was significantly increased while the percentage of fibers between 1500 and 3000 μm^2^ and larger than 3500 μm^2^ was significantly decreased, compared to WT counterparts. NAC treatment reduced the proportion of fibers in the 0–500 μm^2^ interval and increased the proportion of fibers larger than 3500 μm^2^ (Supplementary Material Figure S4A). In contrast, NAC treatment did not significantly affect fiber size distribution in *dy^2J^/dy^2J^* triceps muscle. Furthermore, in 5-week-old *dy^2J^/dy^2J^* quadriceps muscle, the percentage of fibers with cross sectional areas in the range of 0 to 500 μm^2^ was significantly increased while the percentage of fibers in the range of 1000–1500 μm^2^ was significantly decreased. Vitamin E treatment normalized the proportion of small fibers (0–500 μm^2^) in quadriceps muscle (Supplementary Material Figure S4C) but did not affect fiber size distribution in *dy^2J^/dy^2J^* triceps muscles (Supplementary Material Figure S4D).

In summary, these data indicate that NAC and vitamin E treatments both improve *dy^2J^/dy^2J^* skeletal muscle morphology.

### 3.4. Fibrosis is Prevented in NAC Treated dy^2J^/dy^2J^ Muscles

**Figure 4:**
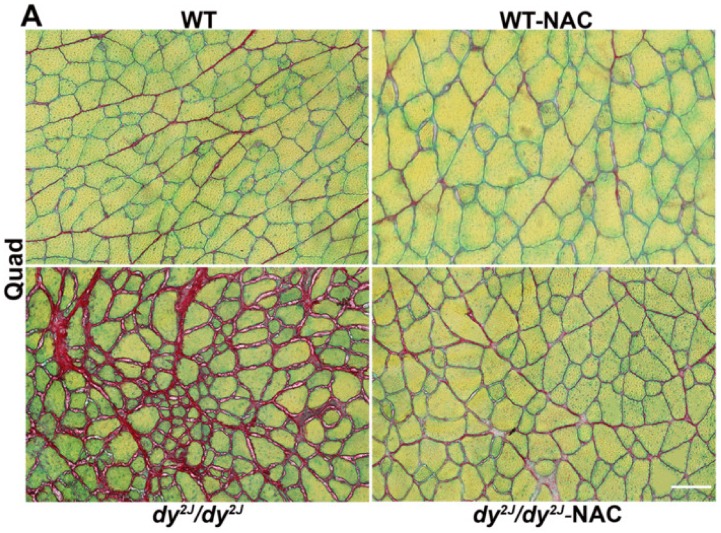

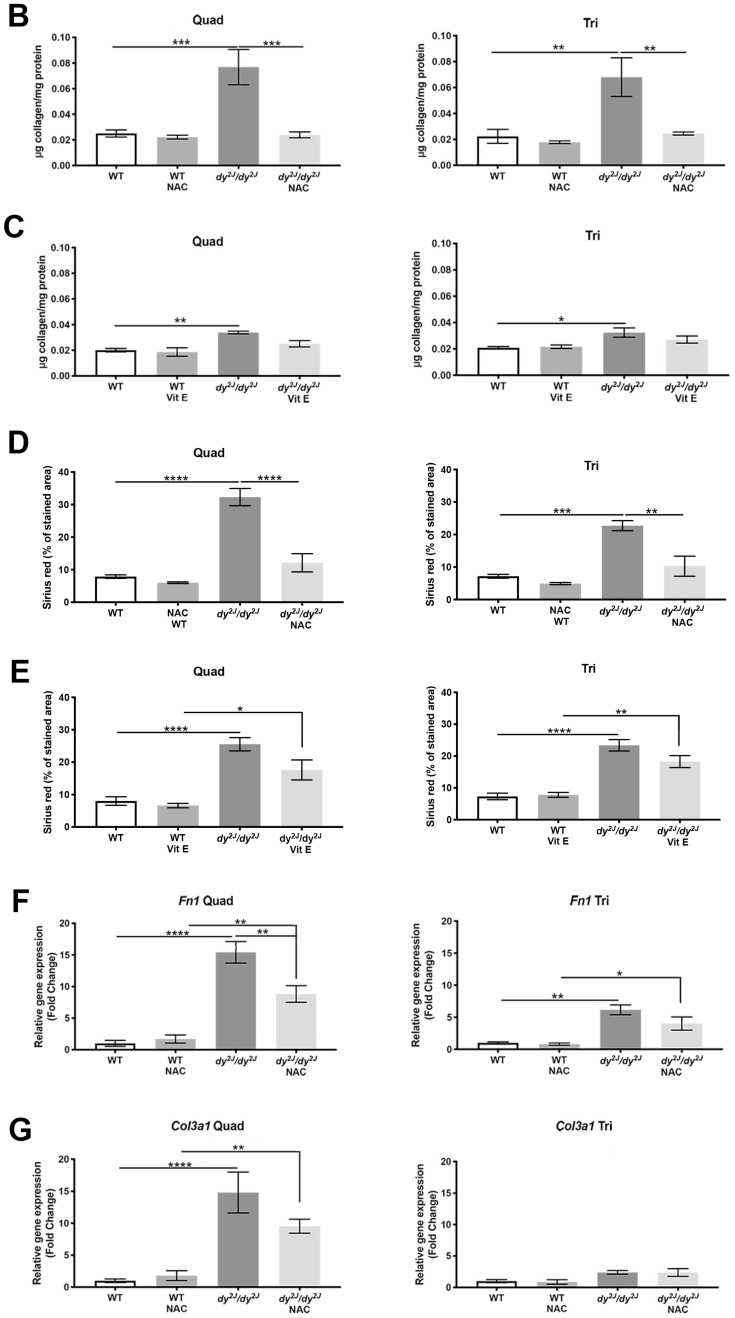
(**A**) Representative Fast Green and Sirius Red-stained quadriceps muscle sections. Bar: 100 µm; (**B**) Collagen content in 6 WT, 10 WT NAC, 8 *dy^2J^/dy^2J^*, 6 *dy^2J^/dy^2J^* NAC quadriceps and 5 WT, 10 WT NAC, 7 *dy^2J^/dy^2J^*, 6 *dy^2J^/dy^2J^* NAC triceps muscles; (**C**) Collagen content in 5 WT, 6 WT vit E, 5 *dy^2J^/dy^2J^*, 5 *dy^2J^/dy^2J^* vit E quadriceps and triceps muscles. Quantitative analysis of collagen content (fibrosis area) in (**D**) 4 WT, 5 WT NAC, 5 *dy^2J^/dy^2J^*, 4 *dy^2J^/dy^2J^* NAC quadriceps and 4 WT, 4 WT NAC, 3 *dy^2J^/dy^2J^*, 3 *dy^2J^/dy^2J^* NAC triceps muscles and (**E**) 5 WT, 4 WT vit E, 5 *dy^2J^/dy^2J^*, 4 *dy^2J^/dy^2J^* vit E quadriceps and 4 WT, 4 WT vit E, 4 *dy^2J^/dy^2J^*, 3 *dy^2J^/dy^2J^* vit E triceps muscles; (**F**,**G**) qPCR analysis of genes related to fibrotic tissue build-up; (**F**) Expression of *Fn1* encoding fibronectin in quadriceps and triceps muscles; (**G**) Expression of *Col3a1* encoding the α1-chain of collagen III in quadriceps and triceps muscles. Results are expressed as mean ± SEM and are expressed as fold change of WT in 5 WT, 5 WT NAC, 4 *dy^2J^/dy^2J^* and 6 *dy^2J^/dy^2J^* NAC. * *p* < 0.05, ** *p* < 0.01, *** *p* < 0.001 and **** *p* < 0.0001.

One of the typical characteristics of laminin α2 chain-deficiency is pathological fibrosis, which has been shown to be increased 2.5-fold in *dy^2J^/dy^2J^* quadriceps and triceps muscles compared with wild-type muscles [32]. We measured fibrosis by both morphometric and colorimetric evaluation of Fast Green and Sirius Red-stained sections. Strikingly, NAC treatment prevented the development of fibrosis in *dy^2J^/dy^2J^* in both quadriceps and triceps muscles (Figure 4A,B,D). Vitamin E treatment, however, did not prevent fibrosis development in *dy^2J^/dy^2J^* quadriceps and triceps muscles (Figure 4C,E, Supplementary Material Figure S5).

To further analyze connective tissue infiltration, we measured the relative gene expression of fibrosis-related genes *Fn1* (encoding fibronectin) and *Col3a1* (encoding the α1 subunit of collagen III) in quadriceps and triceps muscles. We found that the expression of *Fn1* was significantly increased in *dy^2J^/dy^2J^* quadriceps and triceps muscles. NAC treatment significantly inhibited the upregulation of *Fn1* expression level in quadriceps but not in triceps muscle (Figure 4F). *Col3a1* gene expression was also greatly increased in *dy^2J^/dy^2J^* quadriceps muscle but not in *dy^2J^/dy^2J^* triceps muscle. NAC-treatment only slightly (but statistically non-significant) prevented the upregulation of *Col3a1* expression level in *dy^2J^/dy^2J^* quadriceps muscle (Figure 4G). Unlike NAC, vitamin E treatment did not modulate gene expression of *Fn1* or *Col3a1* in *dy^2J^/dy^2J^* quadriceps muscle (data not shown).

In short, these data show that NAC in particular prevents fibrosis in treated *dy^2J^/dy^2J^* skeletal muscle.

### 3.5. Inflammation and Increased ROS Levels are Prevented in Response to NAC and Vitamin E

**Figure 5:**
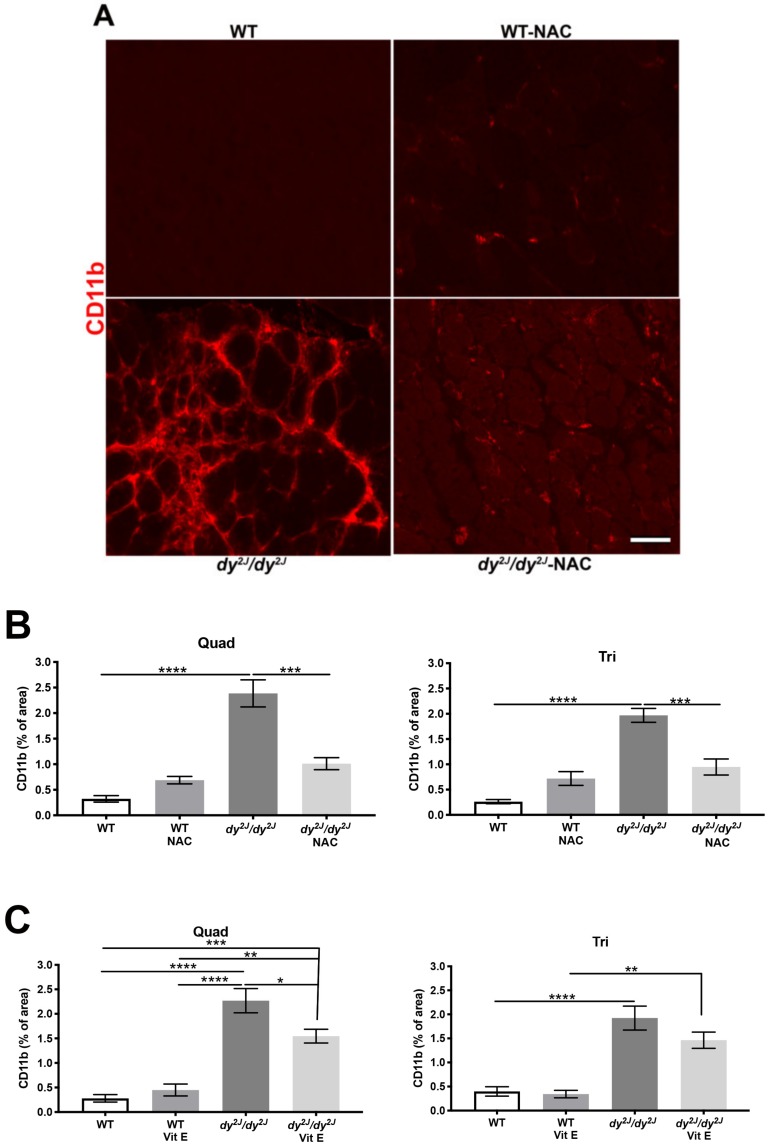
(**A**) Representative CD11b-stained quadriceps muscle sections; (**B**) Quantification of CD11b-stained areas in 7 WT, 9 WT NAC, 5 *dy^2J^/dy^2J^*, 6 *dy^2J^/dy^2J^* NAC quadriceps and triceps muscles; (**C**) CD11b-positive areas in 5 WT, 6 WT vit E, 5 *dy^2J^/dy^2J^*, 5 *dy^2J^/dy^2J^* vit E quadriceps and triceps muscles. * *p* < 0.05, ** *p* < 0.01, *** *p* < 0.001 and **** *p* < 0.0001. Bar: 50 μm.

Since inflammation is recognized as a critical driver of disease pathology in many muscular dystrophies including LAMA2-CMD [34,35], we assessed the inflammatory response in treated and non-treated animals. We observed a significant increase of the CD11b-positive area (macrophages, monocytes, NK cells and granulocytes) in 5- and 6-week-old *dy^2J^/dy^2J^* quadriceps and triceps muscle (Figure 5A–C, Supplementary Material Figure S6).

Notably, NAC significantly inhibited the development of CD11b-positive areas in quadriceps and triceps muscles (Figure 5A,B). Vitamin E also prevented inflammation, but only in quadriceps muscle (Figure 5C, Supplementary Material Figure S6).

**Figure 6:**
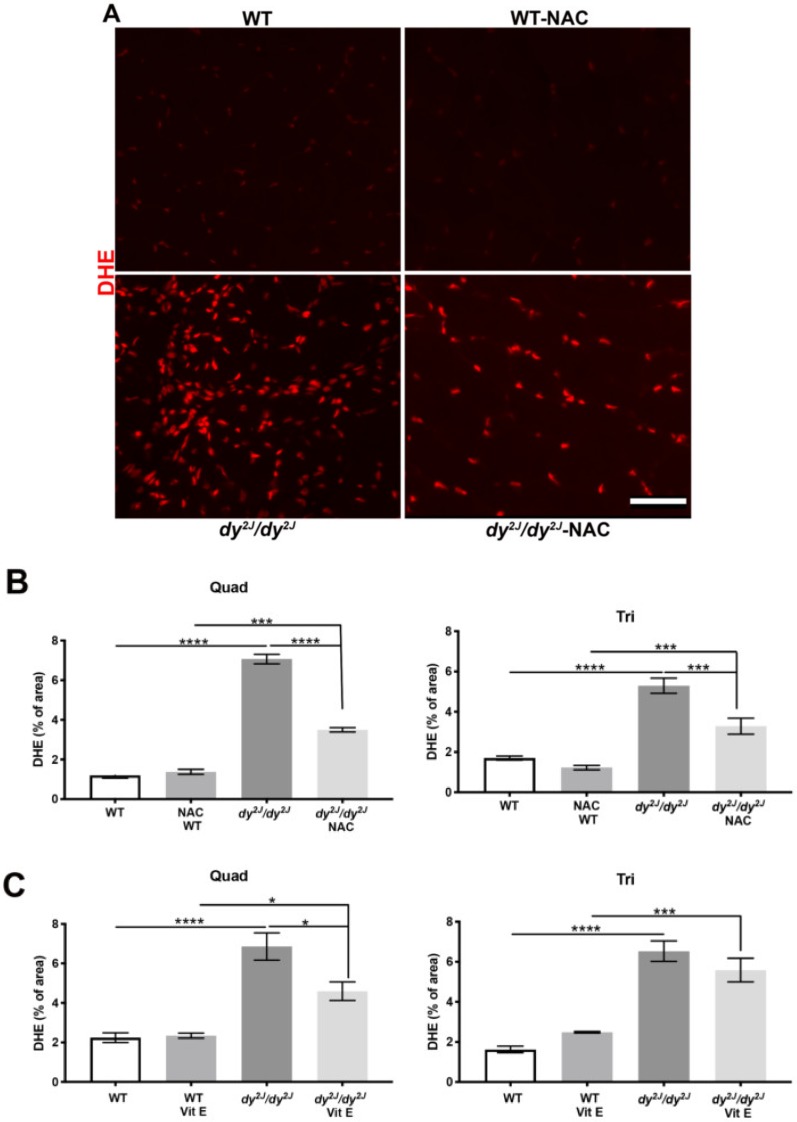
(**A**) Representative DHE-stained quadriceps muscle sections; (**B**) Quantification of DHE-positive areas in 6 WT, 6 WT NAC, 5 *dy^2J^/dy^2J^*, 6 *dy^2J^/dy^2J^* NAC quadriceps and triceps muscles; (**C**) DHE-positive areas in 6 WT, 5 WT vit E, 5 *dy^2J^/dy^2J^*, 5 *dy^2J^/dy^2J^* vit E quadriceps and triceps muscles. * *p* < 0.05, *** *p* < 0.001 and **** *p* < 0.0001. Bar: 50 μm.

ROS levels (detected by DHE staining) were also enhanced in 5- and 6-week-old *dy^2J^/dy^2J^* quadriceps and triceps muscle and NAC treatment significantly decreased the DHE-positive area in both quadriceps and triceps muscles (Figure 6A,B). Additionally, vitamin E treatment inhibited ROS formation in quadriceps but not in triceps muscles (Figure 6C, Supplementary Material Figure S7).

Altogether, these data imply that both NAC and vitamin E are able to prevent inflammation and ROS production in *dy^2J^/dy^2J^* skeletal muscles.

We also assessed the expression of antioxidant-related genes including *Gclc*, encoding glutamate-cysteine ligase catalytic subunit; *Hmox1*, encoding heme oxygenase-1; *Nqo1* encoding NAD(P)H quinone oxidoreductase and *Txn1*, encoding thioredoxin reductase 1. *Gclc* expression was significantly reduced in both 6-week-old *dy^2J^/dy^2J^* quadriceps and triceps muscle and NAC significantly restored *Gclc* expression in *dy^2J^/dy^2J^* quadriceps muscle (and to a lesser extent in triceps). *Gclc* expression was also reduced in 5-week-old *dy^2J^/dy^2J^* quadriceps and a similar trend was seen in *dy^2J^/dy^2J^* triceps. However, vitamin E treatment did not increase *Gclc* expression significantly. *Hmox1* expression showed a trend toward reduction in 6-week-old *dy^2J^/dy^2J^* triceps muscles and NAC slightly increased the expression. *Nqo1* expression was significantly reduced in 6-week-old *dy^2J^/dy^2J^* triceps muscle and NAC slightly (but statistically non-significant) increased *Nqo1* expression. Finally, *Txn1* expression did not differ between the groups (Supplementary Material Figure S8).

### 3.6. Apoptosis is Prevented upon NAC Treatment

**Figure 7:**
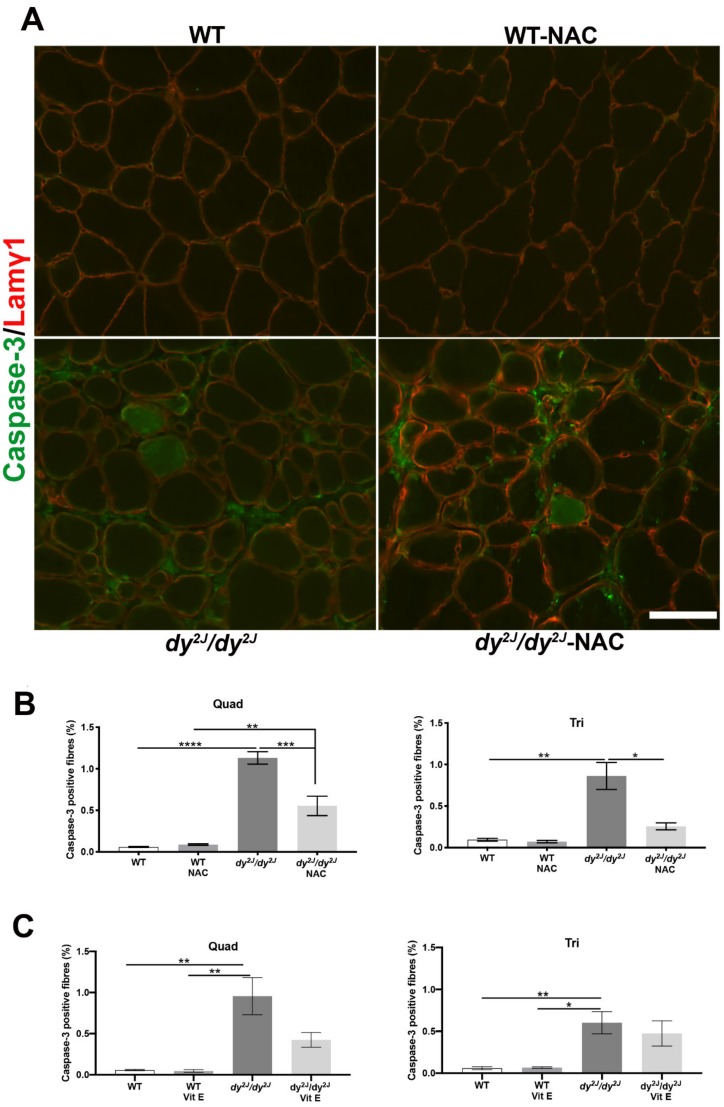
(**A**) Representative caspase-3-stained quadriceps muscle sections; (**B**) The number of myofibers positively stained for caspase-3 (green color) in 5 WT, 6 WT NAC, 7 *dy^2J^/dy^2J^*, 5 *dy^2J^/dy^2J^* NAC quadriceps and 5 WT, 5 WT NAC, 9 *dy^2J^/dy^2J^* and 5 *dy^2J^/dy^2J^* NAC triceps muscles; (**C**) The number of muscle fibers positively stained with caspase-3 (green color) in 5 WT, 4 WT vit E, 5 *dy^2J^/dy^2J^*, 4 *dy^2J^/dy^2J^* vit E quadriceps and 5 WT, 4 WT vit E, 5 *dy^2J^/dy^2J^* and 4 *dy^2J^/dy^2J^* vit E triceps muscles. * *p* < 0.05, ** *p* < 0.01, *** *p* < 0.001 and **** *p* < 0.0001. Bar: 50 μm.

As apoptosis contributes to the disease progression of LAMA2-CMD [36,37], we analyzed the number of apoptotic fibers in skeletal muscle sections. The number of caspase-3- positive fibers (expressing caspase-3 and pro-caspase-3 proteins) was significantly increased in both quadriceps and triceps muscle of 5- and 6-week-old *dy^2J^/dy^2J^* mice (Figure 7A–C). NAC treatment significantly reduced the number of positively stained fibers in both muscles (Figure 7A,B) while vitamin E treatment only slightly decreased the amount of caspase-3-positive fibers in *dy^2J^/dy^2J^* quadriceps muscle (Figure 7C).

## 4. Discussion

Despite significant development of successful genetic and pharmacological preclinical treatment strategies in mice there is still no cure for LAMA2-CMD, the second-most common form of congenital muscular dystrophy [1,38,39]. We here demonstrate increased ROS production in LAMA2-CMD mouse and patient skeletal muscle. Patients with complete laminin α2-deficiency show larger DHE-positive areas in skeletal muscle as opposed to patients with partial laminin α2-deficiency. In general, patients with complete deficiency have a more severe form of LAMA2-CMD [36] and thus, a correlation between the ROS levels and severity may exist although more samples would be required to confirm this hypothesis.

We further show that separate treatment with two antioxidant drugs (both approved for human use) prevents muscular dystrophy progression in the *dy^2J^/dy^2J^* mouse model of LAMA2-CMD. NAC has been shown to be a very effective antioxidant preventing respiratory muscle weakness and fatigue following exposure to chronic sustained hypoxia [40] and chronic intermittent hypoxia [41] in mice and rats. Administration of NAC also led to a decrease in oxidative stress markers including protein carbonylation and improved the cardiac function in a mouse model of LMNA cardiomyopathy [42]. Additionally, several studies show the benefits of NAC as a potential therapeutic treatment for Duchenne muscular dystrophy, using dystrophic dystrophin-deficient *mdx* mice [43,44,45,46]. However, frequent side effects of NAC comprise nausea and vomiting when taken orally [47]. Indeed, previous studies reported significantly lower body weights (*mdx* and C57 mice) and reduced liver (C57 mice) and muscle weights (*mdx* mice) in NAC-treated mice, which could arise from cysteine toxicity [44]. We administered NAC with IP-injections and did not observe reduced body weight or muscle weight but a significantly lower liver weight of NAC-treated WT mice (but not in NAC-treated *dy^2J^/dy^2J^* mice).

Moreover, we demonstrate that NAC treatment improves grip strength and morphological features in skeletal muscles as well as preventing the development of fibrosis, inflammation and ROS levels increase in *dy^2J^/dy^2J^* mice. One feature that was not improved by NAC was the number of stand-ups and this may be due to the fact that NAC only had little effect on the peripheral nervous system in *dy^2J^/dy^2J^* mice. In patients, mild neuropathic changes may be detected [35] but nerve conduction velocities are also normal in some cases of LAMA2-CMD. Nonetheless, we propose that NAC is a potential therapeutic strategy for LAMA2-CMD.

Clinical data on the effects of NAC on skeletal muscle are available. For example, intravenous infusion of NAC inhibited fatigue development in tibialis anterior muscle during repetitive, low frequency electrical stimulation in healthy individuals [48]. Importantly, in a very recent randomized, double-blinded, placebo-controlled trial, NAC was shown to be safe and well-tolerated in individuals with ryanodine receptor 1-related myopathies. NAC did not decrease the oxidative stress in these patients but showed clinically improved physical endurance. The dose in individuals with RYR1-RM was 2,700 mg/d for adults and the pediatric dose was 30 mg/kg/d [49]. *Dy^2J^/dy^2J^* mice received 150 mg/kg/d but it should be pointed out that the optimal dose of NAC is unclear, and studies may show equal efficacy at lower doses or greater efficacy at higher doses [50]. Lastly, a Phase Pilot Trial II-III with NAC in SEPN1-related myopathy was initiated in 2015 [51] but the results of this trial (SELNAC NCT02505087) have not been reported yet.

Vitamin E is a lipid-soluble nutrient with potent phospholipid-directed antioxidant activity [52] and is also considered to be a cytoprotective factor in preventing inflammatory and degenerative processes [53]. Interestingly, vitamin E had some beneficial effects (reduced central nucleation, prevented inflammation and ROS levels increase) in *dy^2J^/dy^2J^* quadriceps muscles but some features such as fibrosis and grip strength were not affected. Thus, NAC-treatment had more profound effects than vitamin E. Vitamin E supplementation has also been evaluated in dystrophin-deficient mice. A two-week supplementation of vitamin E in *mdx* mice diminished inflammation and oxidative stress in diaphragm muscle [31]. In 1960 there was a clinical trial of high dosage of vitamin E in human muscular dystrophy patients, but it was concluded that vitamin E did not produce a positive response compared with placebo [54]. In contrast, a double-blind randomized controlled clinical trial showed that vitamin E, vitamin C, selenium and zinc supplementation improved skeletal muscle function by reducing oxidative stress and enhancing the antioxidant defenses in patients with facioscapulohumoral dystrophy [55].

Several other compounds already approved for human use have been evaluated in different mouse models of LAMA2-CMD and include for example omigapil (tested in *dy^W^*/*dy^W^* and *dy^2^^J^*/*dy^2^^J^* mice) [56]; doxicycline (tested in *dy^W^*/*dy^W^*) [57]; bortezomib (tested in *dy^3K^*/*dy^3K^* and *dy^2^^J^*/*dy^2^^J^*) [22,35]; losartan and a losartan-derivative (tested in *dy^W^*/*dy^W^* and *dy^2^^J^*/*dy^2^^J^*) [58,59,60]; metformin (tested in *dy^2^^J^*/*dy^2^^J^*) [25], prednisolone (tested in *dy*/*dy*) [61] and clenbuterol (tested in *dy*/*dy*) [62]. It should be noted that these drugs only partially ameliorate disease (although it is incredibly difficult to compare studies that were performed in different mouse models, in different laboratories and with different outcome measures). Similarly, neither NAC nor vitamin E target the primary genetic defect and they are not expected to completely cure LAMA2-CMD. Instead, antioxidant treatment strategies could be used as supportive treatments that may improve many of the pathological symptoms in LAMA2-CMD. Moreover, a combinatorial treatment that works through diverse mechanisms might also prove to be more efficient than any single treatment [63,64,65]. Furthermore, it would be interesting to test NAC in combination with vitamin E and evaluate more long-term effects of the two compounds. It should further be mentioned that some of the drugs mentioned above have limits as pharmaceutical treatment of LAMA2-CMD, since deleterious effects upon long-term administration in humans have been noted (*e.g*., doxycycline and bortezomib). NAC, on the other hand, has been FDA-approved for children since 1963 and has a long-established safety record [66,67] and benefits in a wide range of diseases [68,69,70,71]. Vitamin E also has been also safely used in children with different diseases for years [72,73].

In summary, we demonstrate that NAC treatment preserved muscle strength, reduced central nucleation, apoptosis, inflammation and fibrosis and decreased oxidative stress in LAMA2-CMD muscle. Moreover, vitamin E improved morphological features and reduced inflammation and ROS levels in *dy^2J^/dy^2J^* muscle. We thus recommend evaluating the efficacy and safety of NAC and vitamin E, respectively, in humans with LAMA2-CMD.

## Figures and Tables

**Table 1:** Details of control individuals and patients including biopsy site, age at biopsy, the size of dihydroethidium (DHE)-positive areas in percentage of total section size.

Individual	Muscle	Age at Biopsy	*LAMA2* Mutation	Laminin α2- Deficiency	DHE Positive Area (%)
Patients	Quadriceps	4 years	Unknown	Partial	4.94
	Unknown	17 years	Unknown	Complete	5.27
	Unknown	22 days	Homo c.2208 + 2T>C	Complete	10.55
	Radial forearm	29 years	Homo c.2230C>T	Partial	4.23
	Deltoid	2 years	Unknown	Complete	6.42
	Deltoid	3.5 years	Unknown	Complete	10.27
Controls	Radial forearm	33 years	-	-	3.56
	Quadriceps	17 years	-	-	3.10
	Vastus	2 months	-	-	2.34
	Unknown	2.5 years	-	-	1.88

## Supplementary Materials

The following are available online at https://www.mdpi.com/2076-3921/9/3/244/s1. Figure S1: Representative DHE-stained and 4HNE-stained quadriceps muscle sections, Figure S2: Initial body weight (IBW) and final body weight (FBW) prior and after NAC and vitamin E treatments, Figure S3: Muscle and liver weights after the treatments, Figure S4: Cross-sectional area of quadriceps and triceps myofibers in NAC and vitamin E treated animals, Figure S5–7: Representative Fast Green and Sirius Red-stained, CD11-stained and DHE-stained quadriceps muscle of vitamin E treatment groups, Figure S8: qPCR analysis of genes linked to oxidative stress in quadriceps and triceps muscles.
