# Supplementary material for: Antioxidants Reduce Muscular Dystrophy in the dy2J/dy2J Mouse Model of Laminin α2 Chain-Deficient Muscular Dystrophy
Source: Antioxidants (Basel). 2020 Mar 18;9(3):244. doi: 10.3390/antiox9030244 (PMC7139799; doi:10.3390/antiox9030244)
Supplement: Supplementary file 1 [file antioxidants-09-00244-s001.pdf]

Supplementary Material

Antioxidants reduce muscular dystrophy in the dy^{2J}/dy^{2J} mouse model of laminin α2 chain-deficient muscular dystrophy

Vahid M. Harandi [1], Bernardo Moreira Soares Oliveira [1,2], Valérie Allamand [1,3], Ariana Friberg [1], Cibely C. Fontes-Oliveira [1] and Madeleine Durbeej [1]

[1] Unit of Muscle Biology, Department of Experimental Medical Science, Lund University, Lund, 221 84, Sweden; : vahid.harandi@med.lu.se (V.M.H.); (B.S.O.); (V.A.); (A. F.); (C.C.F.-O.); (M.D.)
[2] Functional Genomics & Metabolism Unit, Department of Biochemistry & Molecular Biology, University of Southern Denmark, Odense, 5230, Denmark (B.S.O)
[3] Sorbonne Université, Inserm, UMRS974, Centre de Recherche en Myologie, Paris, 75013, France (V.A.)
* Correspondence: vahid.harandi@med.lu.se (V.M.H.) Tel.: +46(0) 462220679

TABLE OF CONTENTS

Title

Page

Figure S1. Representative DHE-stained and 4HNE-stained quadriceps muscle sections. **S-2**

Figure S2. Initial body weight (IBW) and final body weight (FBW) prior and after treatments. **S-2**

Figure S3. Muscle and liver weights. **S-3**

Figure S4. Cross-sectional area of quadriceps and triceps myofibers. **S-4**

Figure S5. Representative Fast Green and Sirius Red-stained quadriceps muscle of vitamin E treatment groups. **S-4**

Figure S6. Representative CD11b-stained quadriceps muscle of vitamin E treatment groups. **S-5**

Figure S7. Representative DHE-stained quadriceps muscles of vitamin E treatment groups. **S-5**

Figure S8. qPCR analysis of genes linked to oxidative stress in quadriceps and triceps muscles. **S-6**

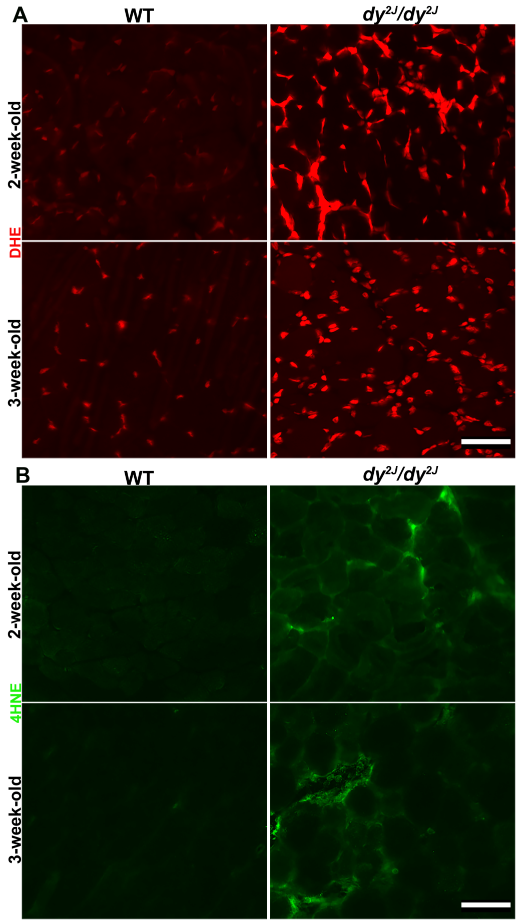

Figure S1. (A) Representative DHE-stained quadriceps muscle sections from 2- and 3-week old animals. (B) Representative 4HNE-stained quadriceps muscle sections from 2- and 3-week-old animals. *P<0.05, **P<0.01, ***P<0.001 and ****P<0.0001. Bars: 50 μm.

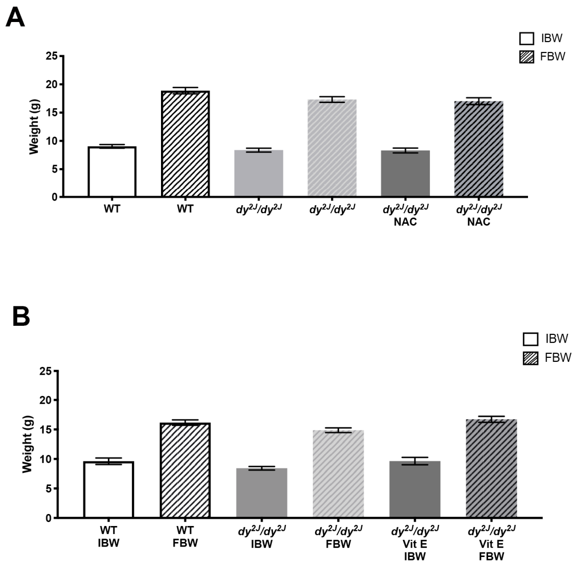

Figure S2. (A) Initial body weight (IBW) and final body weight (FBW) were recorded when the animals were 3-week-old (prior NAC treatment) and 6-week-old (upon termination of NAC treatment), respectively. Results are expressed as mean ± SEM in 10 WT, 11 WT NAC, 5 *dy*^{2J}/*dy*^{2J} and 6 *dy*^{2J}/*dy*^{2J} NAC. (B) IBW and FBW were recorded

when the animals were 3-week-old (prior initiation of vitamin E treatment) and 5-week-old (upon termination of vitamin E treatment), respectively. Results are expressed as mean ± SEM in 12 WT, 7 WT vit E, 5 *dy*^{2J}/*dy*^{2J} and 6 *dy*^{2J}/*dy*^{2J} vit E.

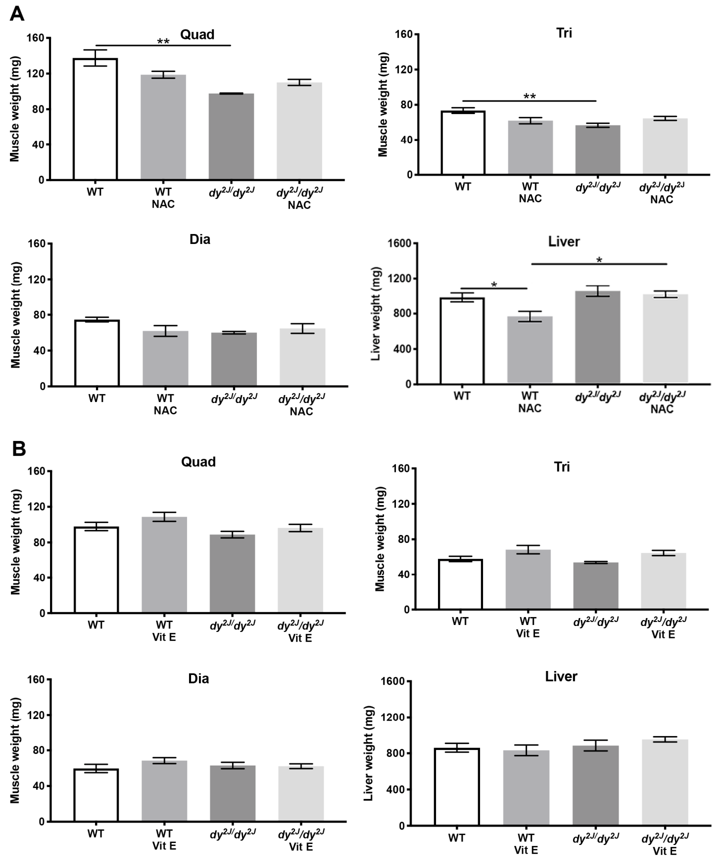

Figure S3. Muscle and liver weights. (A) Quadriceps muscle (Quad), triceps muscle (Tri), diaphragm muscle (Dia) and liver weights. Values are expressed in grams (weights collected after three weeks of NAC treatment) in 8 WT, 8 WT NAC, 5 *dy*^{2J}/*dy*^{2J} and 6 *dy*^{2J}/*dy*^{2J} NAC. (B) Quadriceps, triceps diaphragm muscle and liver weights. Values are expressed in grams (weights collected after two weeks of treatment). Results are expressed as mean ± SEM in 9 WT, 8 WT vit E, 5 *dy*^{2J}/*dy*^{2J} and 5 *dy*^{2J}/*dy*^{2J} vit E. *P <0.05, **P <0.01, ***P <0.001 and ****P <0.0001.

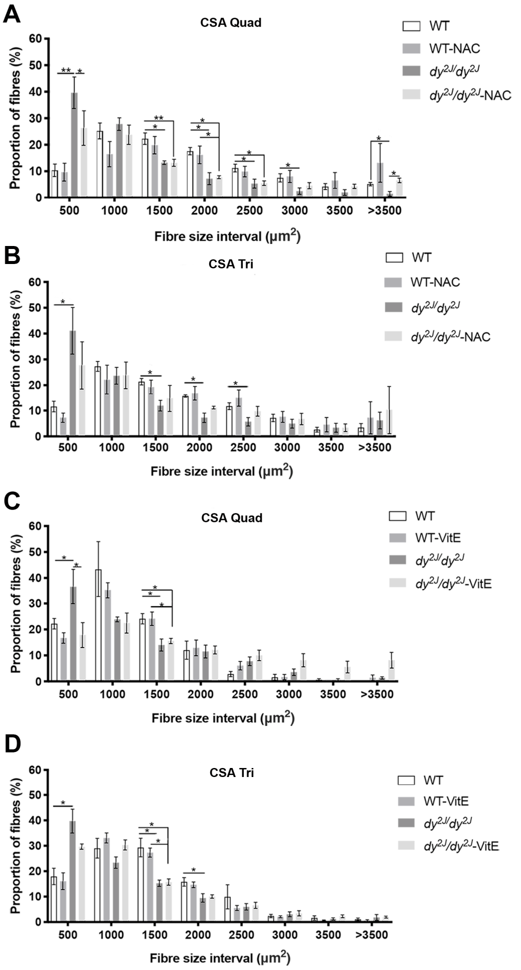

Figure S4. Cross-sectional area of quadriceps (A, C) and triceps (B, D) myofibers. Results are expressed as mean ± SEM in 5 WT, 5 WT NAC, 4 *dy^{2J}/dy^{2J}* , 5 *dy^{2J}/dy^{2J}* NAC and in 5 WT, 6 WT vit E, 5 *dy^{2J}/dy^{2J}* and 5 *dy^{2J}/dy^{2J}* vit E. *P <0.05, **P <0.01, ***P <0.001 and ****P <0.0001.

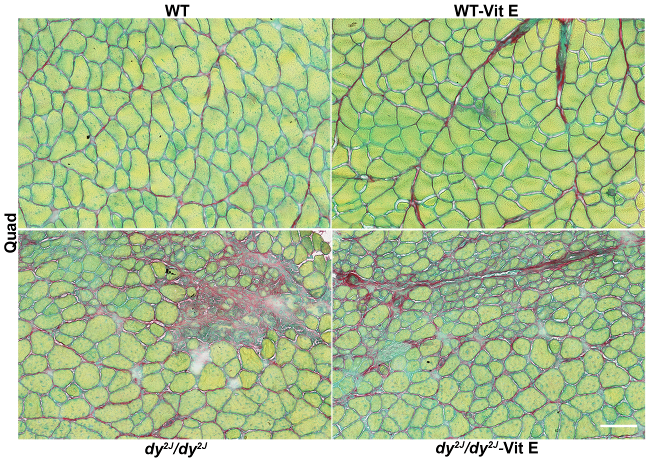

Figure S5. Representative Fast Green and Sirius Red-stained quadriceps muscle of WT, WT vit E, *dy^{2J}/dy^{2J}* and vit E *dy^{2J}/dy^{2J}*. Bar: 100 μm.

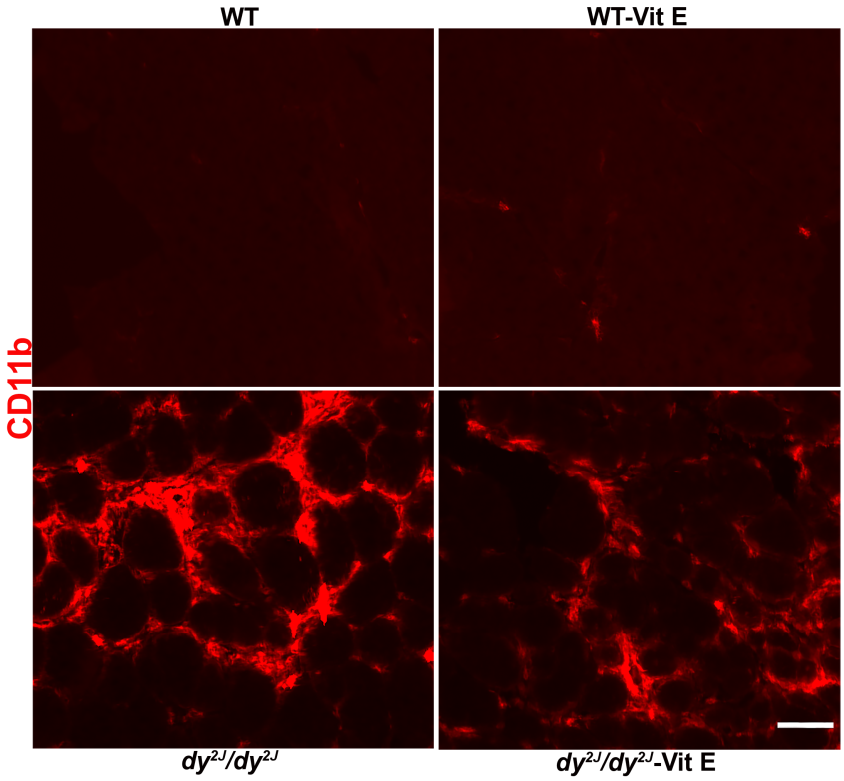

Figure S6. Representative CD11b-stained quadriceps muscle of WT, WT vit E, *dy*^{2J}/*dy*^{2J} and *dy*^{2J}/*dy*^{2J} vit E Bar: 50 µm.

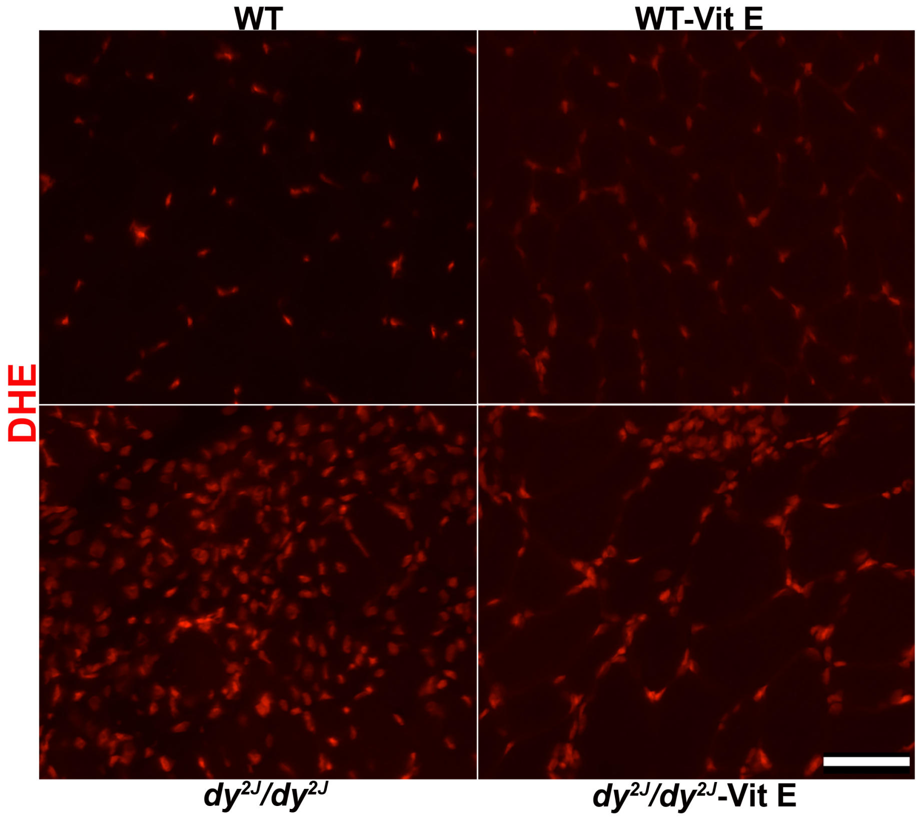

Figure S7. Representative DHE-stained quadriceps muscles of WT, WT vit E, *dy*^{2J}/*dy*^{2J} and *dy*^{2J}/*dy*^{2J} vit E. Bar: 50 µm.

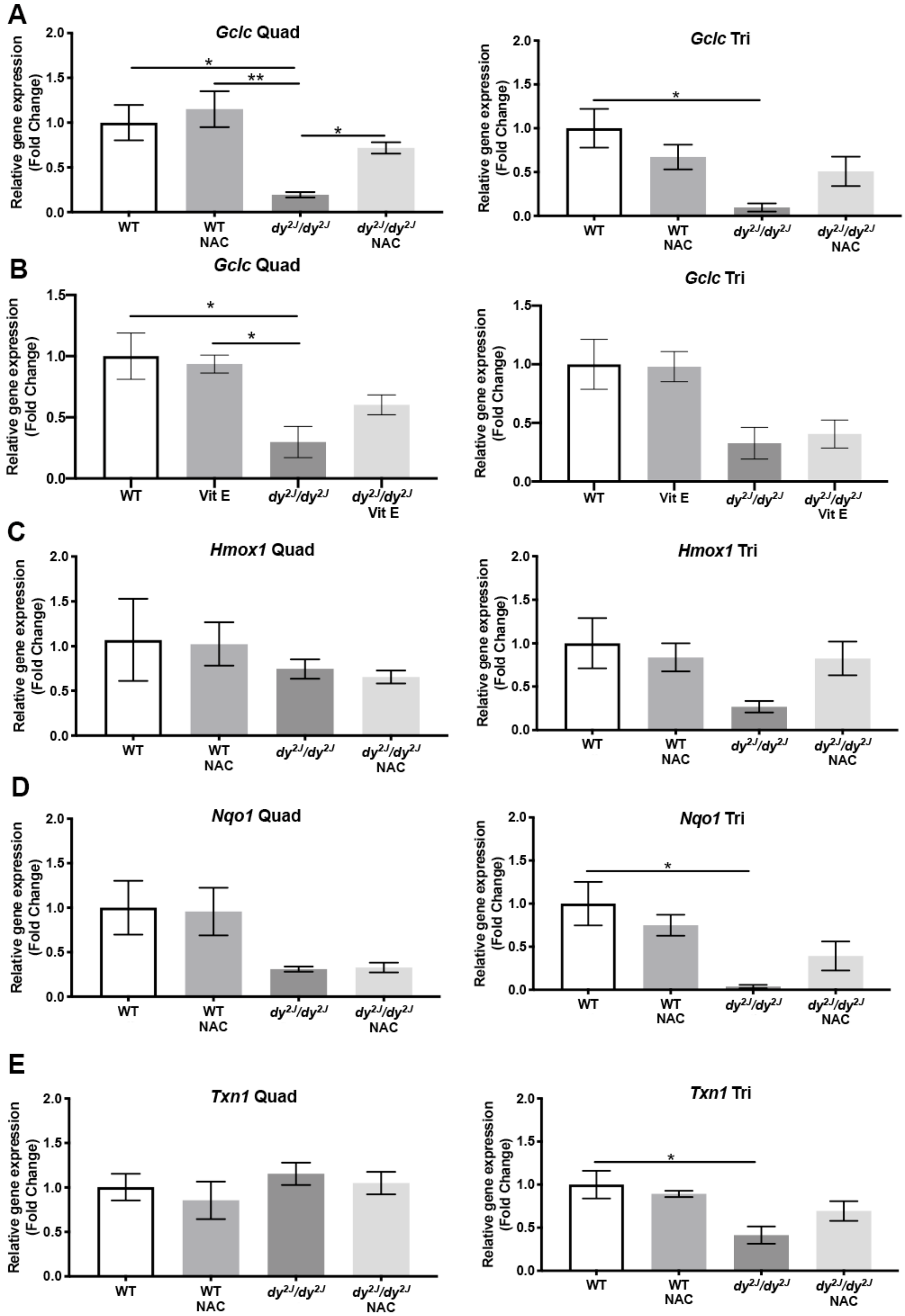

Figure S8. qPCR analysis of genes linked to oxidative stress in quadriceps and triceps muscles. (A, B) Relative gene expression of *Gclc* encoding the glutamate-cysteine ligase catalytic subunit in NAC and vitamin E groups, (C) *Hmox1* encoding pyridine nucleotide oxidoreductases, (D) *Nqo1* encoding quinones to hydroquinones and (E) *Txn1* encodes pyridine nucleotide oxidoreductases. Results are expressed as mean ± SEM and are expressed as fold change of WT in in 5 WT, 5 WT NAC, 4 dy^{2J}/dy^{2J} and 6 dy^{2J}/dy^{2J} NAC. *P <0.05, **P <0.01, ***P <0.001 and ****P <0.0001.
